# Recent Advances in the Science of Burst Wave Lithotripsy and Ultrasonic Propulsion

**DOI:** 10.34133/2022/9847952

**Published:** 2022-02-17

**Authors:** Dima Raskolnikov, Michael R. Bailey, Jonathan D. Harper

**Affiliations:** 1 Department of Urology, University of Washington School of Medicine, Seattle, WA, USA; 2 Center for Industrial and Medical Ultrasound, Applied Physics Laboratory, University of Washington, Seattle, WA, USA

## Abstract

Nephrolithiasis is a common, painful condition that requires surgery in many patients whose stones do not pass spontaneously. Recent technologic advances have enabled the use of ultrasonic propulsion to reposition stones within the urinary tract, either to relieve symptoms or facilitate treatment. Burst wave lithotripsy (BWL) has emerged as a noninvasive technique to fragment stones in awake patients without significant pain or renal injury. We review the preclinical and human studies that have explored the use of these two technologies. We envision that BWL will fill an unmet need for the noninvasive treatment of patients with nephrolithiasis.

## 1. Introduction

Nephrolithiasis—or kidney stone disease—is a common, painful condition that affects nearly 10% of the U.S. population [1]. Stones that form within the renal collecting system are often small enough to pass through the urinary tract spontaneously. However, a growing minority of patients require surgery because of pain, infection, or other sequelae of renal obstruction [2]. Such surgeries involve the minimally invasive fragmentation and extraction of stones, though they can create stone fragments that may lead to additional treatment [3]. For all of these reasons, there has been longstanding interest in noninvasive techniques that facilitate the expulsion of urinary tract stones.

Historical approaches to stone clearance have involved mechanical percussion, inversion, and aggressive diuresis following shock wave lithotripsy [4, 5]. An external physical vibration “lithecbole” showed promise following ureteroscopy [6] and for index distal ureteral stones [7], though this technology has not been widely adopted. Even riding roller coasters appears to have some therapeutic benefit [8]. Ultimately, however, the noninvasive treatment of obstructing urinary tract stones remains an unmet need.

Ultrasound has emerged as a potentially valuable noninvasive tool in this setting for two reasons. First, ultrasonic propulsion may be used to reposition stones throughout the urinary tract, either to facilitate passage or optimize surgical treatment. Second, therapeutic ultrasound in the form of burst wave lithotripsy presents a novel option for the noninvasive fragmentation of stones. The purpose of this review is to describe the history and recent advances in the science of both ultrasonic propulsion and burst wave lithotripsy.

## 2. Background

### 2.1. Shock Wave Lithotripsy (SWL) and Burst Wave Lithotripsy (BWL).

**Figure 1:**
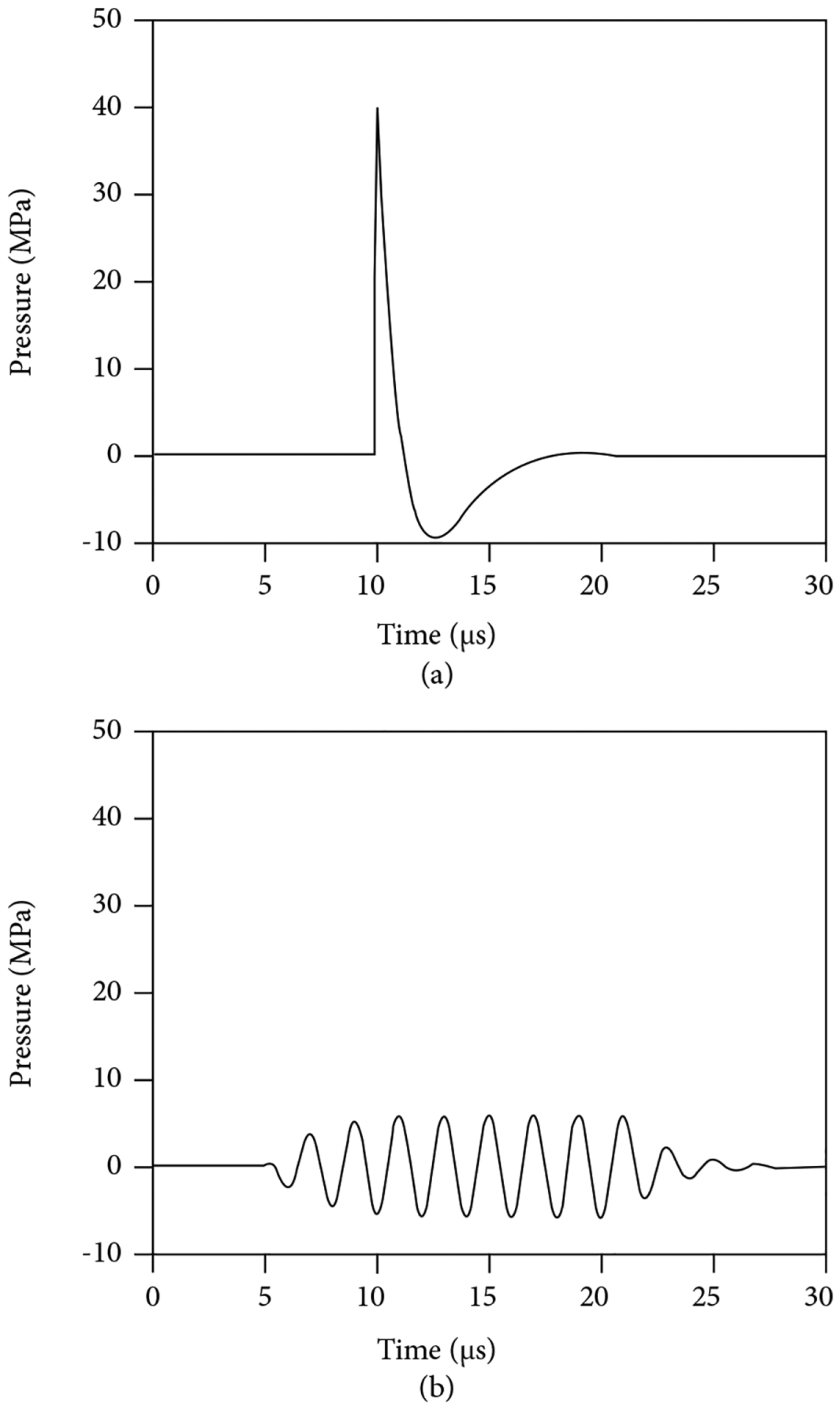
(a) SWL and (b) BWL waveforms.

Shock wave lithotripsy (SWL) is a procedure performed under general anesthesia that involves the administration of single-cycle pulses of energy at a slow rate (≤2 Hz) and high peak pressures (30–100 MPa). This technique—largely patient selection and less so technological advances—has improved considerably since its development by Dornier in 1980, with a better understanding of the importance of skin-to-stone distance, stone density, coupling, shockwave delivery rates, and power ramping [9]. Burst wave lithotripsy (BWL) differs from SWL in that it does not require general anesthesia and may be performed in an ambulatory setting via a handheld device. The pulses generated by SWL and BWL are displayed as a graph of pressure vs. time in Figure 1.

The SWL pulse has one cycle and represents an impulse giving it broadband frequency. The approximately 5 *μ*s SWL pulse repeats every 1/3 to 2 seconds (e.g., at a pulse repetition frequency (PRF) or SW delivery rate of 0.5–3 Hz, resulting in 30–180 shockwaves per minute). The BWL pulse or burst represented in Figure 1 has 8 sinusoidal cycles with *T* indicating the period of one cycle, with an accompanying narrow band frequency. BWL utilizes 10–100 cycles. The center frequency *f* is 1/*T*, and the corresponding angular frequency is *ω* = 2*πf*. BWL has been investigated from 170 to 800 kHz and used at settings of 300–500 kHz in humans. As all waves travel in both time and space, the period *T* is related to the wavelength *λ* by the sound speed *c*; *λ* = *cT*. The PRF for BWL is approximately 10 Hz. The peak negative pressure is clinically relevant as it influences cavitation bubbles and tension in the stone, though pressure polarity can invert upon reflections. The peak negative pressure of SWL is approximately 10 MPa, as compared to 5–8 MPa for BWL. The energy of an acoustic pulse is proportionate to the pressure squared integrated over the pulse duration. The SWL pulse and a BWL pulse with 20 cycles are approximately equal in energy because of the BWL pulses’ longer duration. BWL can thus deliver more energy more quickly, by utilizing a higher PRF or longer cycles. BWL can achieve this higher rate because the peak negative pressure is lower and causes less cavitation, which grows to shield the stone if pulses are too fast [10–12].

**Figure 2:**
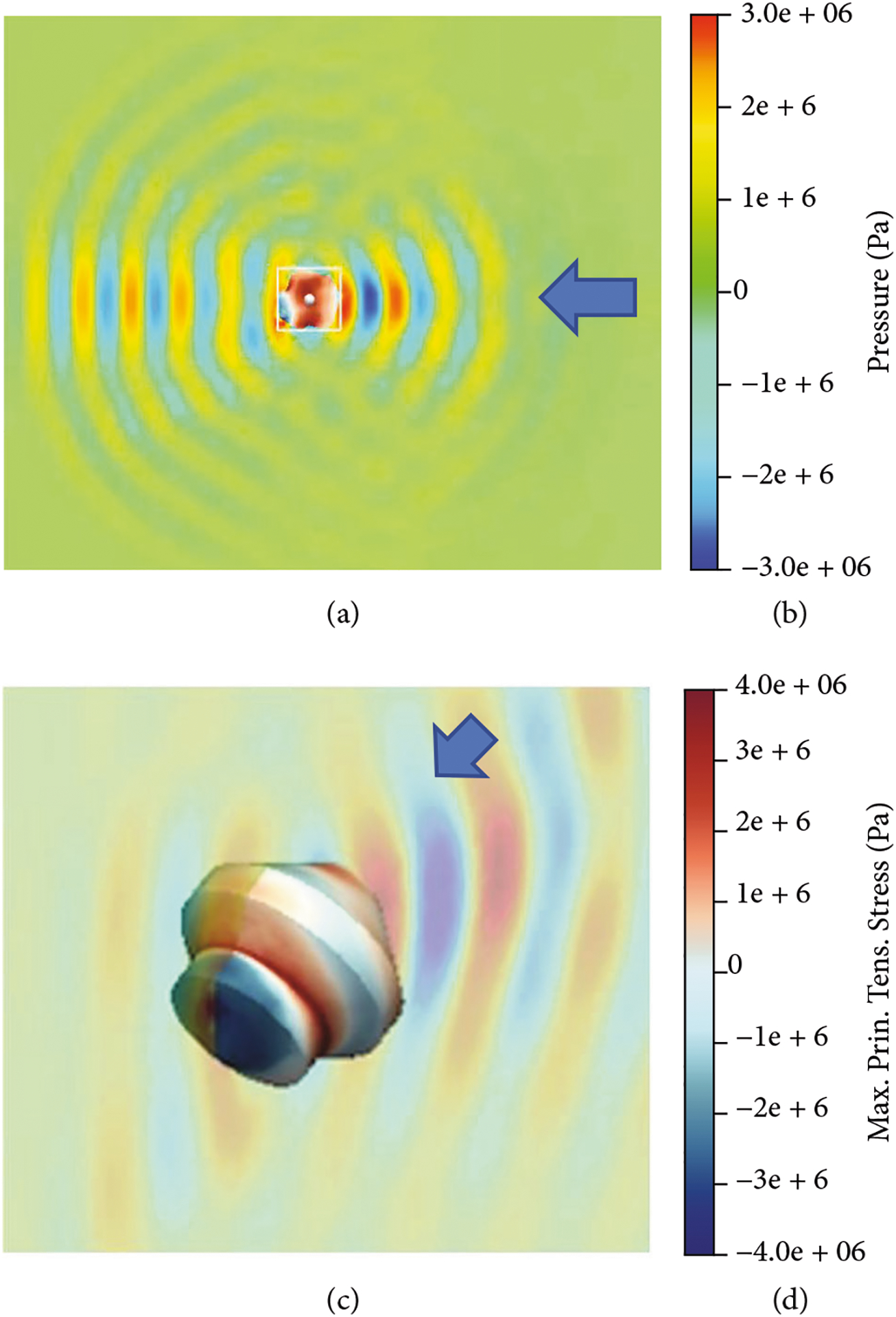
Calculated stress within an irregular 3D stone shape. The axial slice of pressure in the surrounding fluid and stress in the stone ((a) 2.6 mm) is shown zoomed in 3D in (c). The scales (b, d) related to both figures. Arrows indicate the direction of ultrasound propagation (350 kHz). The pressure applied is amplified in the stone, here approximately two times. Above a threshold frequency for a given stone size, amplification is greater than 5 [13] (courtesy of Shunxiang Cao and Tim Colonius, Caltech).

The peak negative pressure of the BWL applied to the stone is thought to be amplified at least 6x within the stone by proper selection of the frequency of stone size [13]. The amplification does not occur with SWL and therefore may help to account for why BWL can have lower pressure than SWL [13]. Amplification occurs for stones over 3 mm for most stone types at frequencies above 300 kHz, but smaller stones and fragments may fragment more effectively and to smaller fragments by completing treatment with higher frequency, such as 800 kHz, according to calculations using a linear elastic model in even irregularly shaped stones as illustrated in Figure 2 [13].

**Figure 3:**
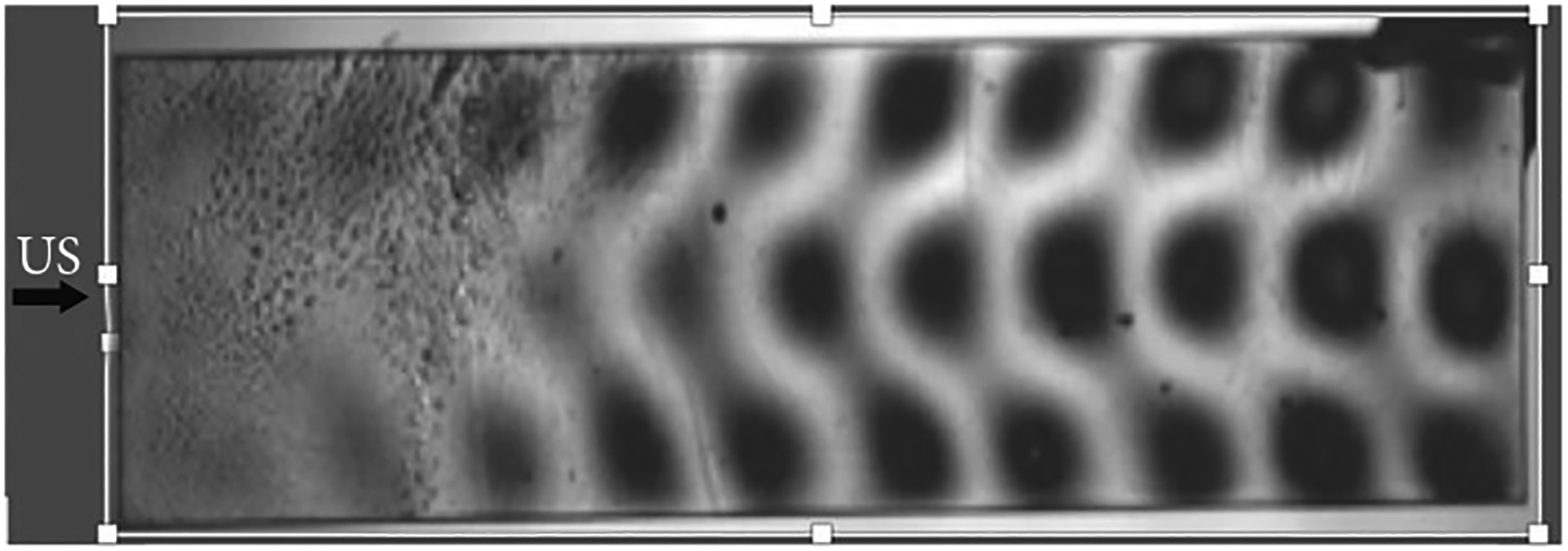
Photoelastic image of stress waves created in a model stone by BWL. Photoelastic imaging reveals the stress (black) of the guided waves in the 5 × 15 mm acrylic bar. The arrow shows the direction of ultrasound propagation (350 kHz). In this larger stone, the multiple-cycle structure of the BWL pulse causes guided waves to develop which cyclically concentrates and amplifies the stress [14] (courtesy of Adam Maxwell, University of Washington).

Measurement and modeling have been compared in model stones where the stresses were visualized within model stones as shown in Figure 3 [14].

This demonstrated that guided waves were developed in the stone. The multiple cycles of the BWL pulse established hot spots of stress that were cyclically loaded and unloaded as the long tone burst reflected within the stone. This demonstrated that many cracks could form throughout the stone at once and that the first fracture appeared at the first internal reflection from the distal end, followed by the next fracture from reflection from the new fractured end. Overall, cracks appeared to form evenly throughout the stone as the stone crumbled with each pulse. The hot spots and cracks are spaced in proportion to the frequency. Therefore, higher frequency (i.e., 800 vs. 400 kHz) can be used to dust stones to <1 mm fragments [13, 15].

### 2.2. Ultrasonic Propulsion.

Ultrasonic propulsion was first described in 2010 by Shah et al. at the University of Washington as a potential tool to reposition stones within the urinary tract [16, 17]. The technology uses transcutaneous ultrasound waves to produce a radiation force on the stone to cause the stone to move in roughly centimeter hops [16]. The team developed a theoretical model to readily calculate this force from an arbitrary acoustic beam on a spherical stone [17], and in general, the force is proportionate to the acoustic power on the stone divided by the speed of sound in the medium around the stone. It turns out even diagnostic ultrasound intensity levels are sufficient to lift a 1 cm stone but the pulse duration and therefore time-averaged intensity needs to be higher to move the stone [18].

Shah et al. [16] produced a novel device to reposition kidney stones using ultrasound radiation force guided by a handheld ultrasound imaging transducer. The effects of nonlinear acoustic propagation and radiation force were calculated [19]. The 2010 device combined a commercial imaging probe (HDI P4-2, Philips Healthcare, Andover, MA) and a focused ultrasound probe, coupled with an HDI 5000 (Philips Healthcare, Andover, MA) to generate ultrasound images. The focused probe consisted of an 8-element annular array with a nominal frequency of 2.0 MHz, activate area diameter of 63 mm, and inner imaging aperture of 20 mm. The focal depth was programmable within a range of 4.5–8.5 cm. Artificial and human kidney stones were implanted into kidney-mimicking phantoms that simulated the renal collecting system; then, focused ultrasound was delivered at instantaneous acoustic power of 5 W-40 W, duty cycle of 50%, and duration of 2–5 sec. While monitoring stones within the kidney phantom by fluoroscopy and video photography, stones were seen to move out of selected locations at velocities of 1 cm/sec. These results suggested that ultrasonic propulsion may be an effective noninvasive tool for helping to render patients stone-free.

The first clinical device was one machine centered on the Verasonics V1 ultrasound engine (Kirkland WA) and used a single diagnostic imaging transducer (C5-2, Philips/ATL, Bothell WA). This was used in the first clinical trial discussed below [20]. Following that trial, the device was refined to provide broader beams and longer duration exposures for ultrasonic propulsion and enabled BWL pulses with the same transducer [21–24]. The iteration of the hand held transducer used in most propulsion and BWL clinical trials to date uses a 350 kHz and 60 mm single element annular element for therapy (H209, Sonic Concepts, Bothell WA) and has within the aperture a phased array transducer (P4-2, Philips/ATL, Bothell, WA) for image guidance. The imaging algorithms utilize features to increase stone contrast including aspects of the twinkling artifact for stone targeting and the distal acoustic shadow for stone size determination [25, 26]. The acoustic outputs are 25 ms pulses at 50% duty cycle at 350 kHz frequency at pulse-averaged intensities of up to 200 W/cm^2^ for 1–3 seconds. The primary skill with propulsion is aligning the force which is away from the transducer in a direction in which the stone or fragment is free to move. Subsequent research studies have investigated “acoustic forceps” using a 256-element array to trap and manipulate stone models through a complex 3D path [27]. Acoustic forceps have been demonstrated to move a glass sphere in a controlled path in the bladder of a porcine model without morphological injury to surrounding tissues [20].

## 3. Ultrasonic Propulsion

Ultrasonic propulsion has been studied extensively in animal models to evaluate efficacy and safety. In 2012, human stones were implanted by retrograde ureteroscopy or ante-grade percutaneous nephroscopy in a live porcine model [28]. The stones were then repositioned with ultrasonic propulsion. Renal tissue was then analyzed histologically, demonstrating no injury at therapeutic levels and only small areas of injury at excessively high energy settings. Following advancements and modifications to the ultrasonic propulsion device, a similar experiment was conducted in 2013 [29]. Calcium stones were inserted via ureteroscopy into 12 porcine kidneys, then pushed via ultrasonic energy. Post-treatment histology again demonstrated no evidence of injury. In 2014, it was demonstrated that a third generation device utilizing ultrasound bursts was equally safe and effective [30]. Connors et al. compared tissue injury generated by ultrasonic propulsion to that of SWL in 3 pig kidneys [31]. On posttreatment histology, no injury was seen using therapeutic ultrasonic propulsion settings. Excessive ultrasonic propulsion treatment parameters generated injury similar to that seen with convention SWL. Wang et al. explored thresholds for tissue injury using ultrasonic propulsion in another model of pig kidneys [32]. The authors found that the maximum spatial peak intensity that was necessary to cause histologically detectable renal injury was sevenfold higher than that seen during transcutaneous treatment.

### 3.1. Clinical Trials.

Human trials of ultrasonic propulsion have now also reported promising results. In 2016, Harper et al. attempted to reposition small stones within the collecting systems of 15 participants [33]. The study population included both awake subjects and those who were anesthetized for endoscopic stone surgeries. Propulsion was successful in 14 of 15 patients, with awake patients describing only rare, mild, and brief discomfort. Clinical benefits included diagnosing fragments versus single stones, facilitating passage of fragments within the kidney, and relief of pain from rotation of a UPJ stone. Dai et al. evaluated ultrasonic propulsion in 18 patients who were undergoing ureteroscopy [34]. Intraoperatively, ultrasonic propulsion was applied transcutaneously, while stone targets were visualized ureteroscopically. Independent review of these videos determined that motion ≥ 3 mm had occurred in 18 of 19 kidneys (95%). Data from the most recent clinical study presented at the 2021 meeting of the American Urological Association suggests that ultrasonic propulsion may help relieve pain and accelerate passage of distal ureteral stones [35], as well as chronic residual fragments [36]. In the case of this latter study, a subject reported never passing fragments following SWL. Eighteen months later, ultrasound propulsion repositioned fragments from the lower pole which he then passed with 2 hours of treatment ending up free of residual fragments [29]. The associated randomized controlled trial of clearing residual fragments is nearing completion.

## 4. Burst Wave Lithotripsy

**Figure 4:**
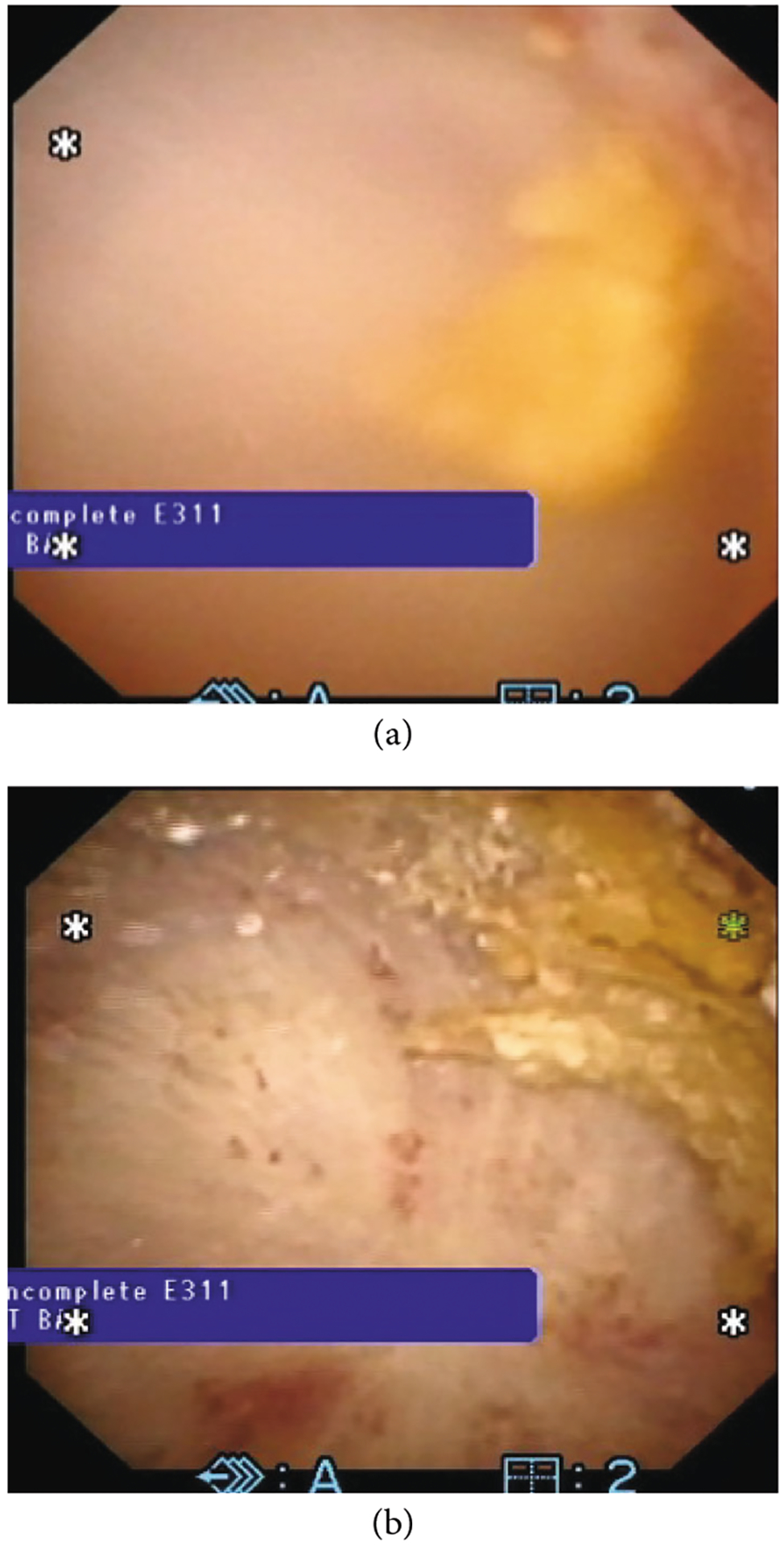
6 mm stone (a) before and (b) after 10 minutes of BWL. Completely broken to <2 mm pieces.

In 2015, Maxwell et al. examined the feasibility of stone fracture by BWL by applying this energy to both artificial and natural calculi *in vitro* [15]. The authors treated stones within a water bath using frequencies of 170 kHz, 285 kHz, and 800 kHz with 3 different transducers. Though treatment time varied by stone composition, exposures at 285 kHz produced only fragments < 2 mm and 800 kHz only <1 mm. The authors concluded that BWL was feasible and that adjustments in ultrasound frequency could help to control stone fragment size (Figure 4). Further refinements in this technique have allowed for the fragmentation of renal stones > 1 cm using a broad beam transducer [37].

Animal studies mirroring those during the development and FDA investigational device exemption phase for ultrasonic propulsion have been completed and also confirmed the safety and efficacy of BWL. May et al. treated 10 pig kidneys with 170 or 335 kHz BWL transducers using variable treatment parameters and while monitoring with real-time ultrasound [38]. Eight kidneys were subsequently evaluated with MRI, while two underwent histologic examination. B-mode echogenicity on ultrasound consistent with cavitation was sensitive and specific for renal injury, though these injuries mirrored those seen with SWL. The authors concluded that real-time US may help to facilitate monitoring of BWL and prevent renal injury. Ramesh et al. tested BWL on 46 human stones within a phantom model of the human kidney, finding that 89% of stones were comminuted within 30 minutes and 70% within 10 minutes of treatment [23]. Incorporating ultrasonic propulsion during a BWL treatment session is thought to provide benefit in determining the endpoint of fragmentation when small stone fragments are cleared from the treatment area. In another study, 7 mm stones were implanted into 3 pig kidneys, where they were then treated with BWL for 30 minutes before nephrectomy [11]. Of the total implanted stone mass, 87% was reduced to <2 mm fragments. There was no gross, histologic, or MRI evidence of renal parenchymal injury. One week survival studies in pig have all resulted in histological findings within normal limits [39].

### 4.1. Clinical Trials.

In 2021, Harper et al. reported the first in-human study of BWL in two patients [24]. In the first subject, BWL was used to target a 7 mm kidney stone during general anesthesia for ureteroscopy. Following 9 minutes of treatment, ureteroscopic examination demonstrated fragmentation to ≤2 mm and only mild petechial hemorrhage. In the second subject, BWL was used to treat a 7.5 mm stone at the ureterovesical junction in an awake patient. The patient tolerated the procedure well, required no anesthesia, and passed the stone on day 15. In another study of 12 subjects reported at the AUA in 2021, BWL during ureteroscopy demonstrated 63% comminution into fragments ≤ 2 mm following 10 minutes of treatment with only mild bleeding on some papillae [40]. Clinical studies are ongoing. Feasibility of fragmentation of kidney stones in the clinic is ongoing with the expectation of moving next to a randomized control trial of treating small 2–7 mm stones as well as treating stones in individuals with spinal cord injury.

## 5. Future Directions

Kidney stone fragmentation and repositioning by BWL and ultrasonic propulsion have shown promising results in preclinical and clinical trials. Based on the initial results, the system was upgraded to enhance imaging, treat at deeper depths, and fragment stones to progressively smaller fragments [41]. A p4-2 style single crystal imaging transducer is used in a central aperture in the therapy probe for imaging guidance and feedback. Commercial imaging probes are rehoused to fit within the therapy transducer. Imaging was further upgraded from a Verasonics Version 1 system to a Vantage research ultrasound engine, which enables harmonic imaging. Resolution and contrast validation supports potentially better resolution of fragments as they break from the stone such that all pieces may be completely treated. Three new therapy transducers were added first to add a higher frequency (800 kHz) to create smaller fragments and transducers at both frequencies with a deeper focus (6 vs. 8 cm). Finally, a new custom class D/E amplifier replaced commercial A/B amplifiers, which allowed more power required by the deeper focus low frequency (400 kHz) transducer, interleaving of propulsion pulses with BWL, longer BWL burst durations [42], and real-time monitoring of the instantaneous and average electrical powers. Interleaving was previously shown to accelerate stone breaking with BWL *ex vivo* [43]. These ongoing enhancements to the current technology will provide capabilities to treat a larger patient population as we begin trials breaking and expelling kidney and ureteral stones in the outpatient setting.

## 6. Conclusion

Ultrasonic propulsion and burst wave lithotripsy offer a powerful, handheld tool to target, break, dislodge, and expel stones and stone fragments from the urinary tract in an ambulatory setting. Additional studies of these novel technologies are underway.
